# Health research in the Western Cape province, South Africa: Lessons and challenges

**DOI:** 10.4102/phcfm.v6i1.698

**Published:** 2014-12-15

**Authors:** Leslie London, Tracey Naledi, Sabela Petros

**Affiliations:** 1 School of Public Health and Family Medicine, Division of Public Health Medicine, University of Cape Town, South Africa; 2 Health Department, Western Cape Government, South Africa

## Abstract

**Background:**

Health research can play a critical role in strengthening health systems. However, little monitoring of health research is conducted in African countries to identify whether research contributes to addressing local health priorities.

**Aim/Setting:**

To review the profile of research on the health service platform in the Western Cape province of South Africa which was approved by the health authorities over the period January 2011 to December 2012.

**Methods:**

Databases held by both the Provincial and City of Cape Town health departments were reviewed. Descriptions of research institution, location of research, topic and funding size and source were analysed.

**Results:**

Of the health research approved in the province, 56% of projects were located on the District Health Services platform and 70% were based in the Cape Metropolitan area. For projects reporting budgetary information, the total funding was US $29.2 million. The primary focus of research was on HIV and tuberculosis (TB), whilst relatively few studies addressed nutrition, mental health or injury and there was little health systems research. Research funding was dominated by very large grants from foreign funders for HIV and/or TB research. South African government sources comprised less than 8% of all health research funding.

**Conclusion:**

There is a partial mismatch of donor funding to local health priorities. Greater focus on neglected areas such as mental health, trauma, nutrition and non-communicable disease, as well as greater investment in health systems research, is needed. Unless governments increase funding for research and a culture of research translation is achieved, health research will have limited impact on both local and national priorities.

## Abstrait

**Recherches en santé dans la Province du Western Cape, Afrique du Sud: Leçons et Défis**.

**Contexte:**

La recherche en santé peut jouer un rôle essentiel dans le renforcement des systèmes de santé. Cependant, il y a peu de suivi sur les recherches en santé dans les pays africains pour déterminer si la recherche contribue à donner suite aux priorités en matière de santé.

**Objectif/ Lieu:**

Revoir la recherche sur la plateforme des services sanitaires dans la province du Western Cape d'Afrique du Sud, approuvée par les autorités sanitaires pour la période allant du mois de janvier 2011 au mois de décembre 2012.

**Méthodes:**

On a révisé les banques de données des départements sanitaires de la Province et de la ville du Cap. On a aussi analysé la description des instituts de recherche, les lieux de recherche, les thèmes et le montant et la source des financements.

**Résultats:**

Parmi la recherche sanitaire approuvée dans la province, 56% des projets des Services de Santé de District, et 70% de ceux-ci se trouvaient dans la zone métropolitaine. Le financement total des informations budgétaires relatives aux projets était de 29.2 millions d'USD. La recherche portait principalement sur le VIH et la tuberculose; alors qu'il y avait relativement peu d’études sur la nutrition, la santé mentale ou les blessures et peu de recherches sur les systèmes de santé. Le financement de la recherche était dominé par les subventions importantes des bailleurs de fonds étrangers pour la recherche sur le VIH/la tuberculose. Les sources provenant du gouvernement sud-africain s’élevaient à moins de 8% de tous les financements de recherche en santé.

**Conclusion:**

Il y a un certain décalage dans le financement des bailleurs de fonds pour les priorités locales en matière de santé. Il conviendrait d'insister d'avantage sur les domaines négligés tels que la santé mentale, le traumatisme, la nutrition et les maladies non contagieuses, et aussi de s'investir plus dans la recherche sur les systèmes de santé. A moins que les gouvernements n'augmentent le financement de la recherche et sa valorisation, la recherche dans le domaine de la santé aura un effet limité sur les priorités locales et nationales.

## Introduction

Health research is critical to human development and well-being. Research can contribute to identifying key health problems, the risks for and associations with health states and mustering the evidence for how to address these problems; to assessing health service and health system performance and identifying and evaluating improvement strategies; to informing appropriate policies and programmes; and to building the knowledge base for improved health outcomes. Health research can tell the public, civil society and health managers how their services are doing and what needs improving.^1–3^ Although the importance of health research for potentially improving health outcomes is undisputed, the extent to which investments in health research deliver desired benefits has been challenged.^4–7^ Concerns have also been expressed regarding whether health research meets local priorities,^6, 8^ most commonly framed through analysis by Burden of Disease (BoD) categories.^9–12^ Moreover, it has been noted that despite the great potential for enhancing health research in the clinical domain in South Africa, the contribution of clinical research has been severely constrained by lack of funding and undermined by the lack of any monitoring and evaluation systems for health research.^1^

Given the importance of health for development, inequities in the distribution of health research benefits and the role of research in strengthening Primary Health Care, an integrated strategy for health research has been elevated in South Africa to a legislative responsibility of the National Health Council.^13^ Accordingly, the *National Health Act* 61 of 2003 provides an institutional and regulatory framework for the undertaking of health research in South Africa, including the establishment of a National Health Research Committee and equivalent provincial structures charged with, amongst other responsibilities, identifying research priorities in the country.^3^ Moreover, strengthening of research and development is one of 10 National Ministerial priorities.^14^

In the Western Cape province, the process of oversight and facilitation of health research under the Provincial Health Research Committee involves approval by the Provincial and City of Cape Town (CoCT) health authorities of every research study conducted within their services. In submitting applications to conduct research on the health platform to the authorities, researchers are expected to provide information in their proposals on their projects, to the extent that the services can evaluate whether permission should be given for the research to go ahead. This provides a potential mechanism by which health services can maintain a database of research in the health services, be informed of research findings and make better use of research outputs to inform policy, programmes and practice. Even though the HIV programme in particular has provided very good recent examples of evidence-based policy development,^15^ the culture of using evidence more generally to inform decision making in South African health services is weak.^3^

Given calls at the National Health Summit convened to improve the monitoring and evaluation of the performance of the health research system in South Africa, this article presents a review of research by health services in the Western Cape in 2011 and 2012. It aims to describe the location, type and amount of funding for research as well as the institutional affiliation of researchers and funding sources available for health research in the province.

## Research methods and design

Databases created and maintained by health authorities in the province for research approval were assembled and reviewed for studies submitted and approved between January 2011 and December 2012. The primary database was that held in the Health Research sub-Directorate of the Health Impact Assessment Directorate in the Department of Health of the Western Cape Government for research conducted on the Provincial District Health Service (DHS) platform. This database captures information through mandatory fields in the application form on study topic and type, funding source, research budget, principal investigator affiliation and location of study (facility, district and subdistrict). The databases of the CoCT Health Department and from four tertiary and psychiatric hospitals (Tygerberg, Groote Schuur, Valkenberg and Stikland hospitals) were also reviewed, but provided data only for the 20 months from January 2011 to August 2012. These databases contained limited information on topic and location but no details about funding or investigator affiliation. At the time of conducting the study, there was no consistent content, structure and field format for the different databases. Moreover, health research conducted outside of provincial and municipal health services was not captured in any health service database.

Based on the study title, the topic was post-coded to a variable that included the main BoD categories (HIV, tuberculosis [TB], injury and/or violence, women's health, child health, non-communicable disease, mental health and nutrition), supplemented by coding for a category ‘Other’ (which included disease entities not contained in the main BoD categories) and ‘General’ (which included health system and service issues not specific to a disease or BoD category). The latter included, for example, studies of human resource issues, quality of care and policy studies. Up to three codes for topic were permitted per study. Analysis of study topic included studies conducted in central hospitals as well as on the District and Regional platform for the period January 2011 to August 2012.

For type of study, a descriptor was created for health services research based on the reviewer's assessment of whether the research topic had direct implications for health service development. A second variable was created to code for drug trials.

Investigator affiliation was coded as the South African institution (university or research council) to which the investigator was affiliated or, if the investigator was a non-national, was coded as ‘International’. All investigators based in pharmaceutical companies or private Clinical Research Organisations (CROs) were coded in a single category of ‘Pharma’.

Source of funding was coded as listed in Table 1. The main categories were South African (SA) Government, United States (US) Government, Global Agencies, Other International, University, Health Services, Self-funded and Other. Donor grants directed through government were counted as government funding. Those who did not declare their source of funding were reported as ‘Unknown’.

**TABLE 1:** Coding of funding sources for health research in the Western Cape, 2011–2012.

Category	Includes
SA (South African) Government	All funding from South African government, whether directly through government departments or through research councils such as the Medical Research Council (MRC), Human Sciences Research Council (HSRC) and the National Research Foundation (NRF).
US (United States) Government	Centre for Disease Control (CDC), The U.S. President's Emergency Plan for AIDS Relief (PEPFAR), United States Agency for International Development (USAID), the National Institutes of Health (NIH) and any NIH institute.
Global Agencies	Denotes funding from Foundations (such as AERAS Global TB vaccine foundation, or the Bill & Melinda Gates Foundation) and the Joint United Nations Programme on HIV/AIDS (UNAIDS).
Other International	a. A non-South African university.
	b. International agencies other than US agencies such as the International Development Research Centre (IDRC), Canadian Institute for Health ResearchICIHR), European Union (EU), Wellcome Trust, South African and Netherlands Research Programme on Alternatives in Development (SANPAD), WorldHealth Organisation (WHO), World Dental Federation (FDI) and the European and Developing Countries Clinical Trials Partnership (EDCTP).
Pharma	Private sector pharmaceutical companies.
Foundation	South African Foundations such as the Harry Crossley Foundation.
University	Researchers who indicated their own universities as the source of funding.
Health Services	Researchers who were service providers conducted research funded from within the services.
Self-funded	Researchers who indicated they were unfunded or funded their own research out of their own resources.
Other	a. Local Non-Government Organisations (NGOs): Hospice, ANOVA, Impumelelo, CANSA.
	b. International NGOs working in South Africa: Médecins Sans Frontierès (MSF), Management Sciences for Health (MSH).

Although projects may have received funding from more than one source, only one code was allocated for funding source as the information supplied did not allow for disaggregation of how much funding came from different funders. Where there was any government funding for a study, we coded the funder as ‘SA Government’, thereby making the code ‘SA Government’ as the ‘trump’ code for multiple funders.

Size of budget was based on self-reported budget provided by the researcher in the application documents. Budgets are reported in US$ with a conversion rate from SA currency (ZA Rands) of $1 to ZAR10, the rate prevalent at September 2012. Where researchers failed to report budget or reported budget in unmanageable terms, the data were treated as incomplete.

## Results

During the 20-month period January 2011 to August 2012, a total of 615 projects were approved in the province. Just over half (56%) were projects approved on the DHS platform, whilst the rest were projects implemented at specialist or psychiatric hospitals.

Amongst the 341 projects approved on the DHS platform between January 2011 and December 2012, approximately 50% were projects spanning multiple districts: 72% were located in the Cape Metro, with fewer in other districts (23% in the Cape Winelands; 11% in Eden, 8% in the Overberg; 6% on the West Coast and 4% in the Central Karoo). Within the Cape Metro, 18% of projects were not specific to subdistricts or were located in central, regional, TB, rehabilitation and psychiatric hospitals. Across subdistricts, projects were approximately evenly distributed by subdistrict (Eastern and Khayelitsha [30%]; Tygerberg and Northern [33%]; Klipfontein and Mitchells Plain [35%]; and Southern and Western [36%]). Once studies in the central hospitals were included, over 85% of research studies submitted for approval between January 2011 and August 2012 were located in the Cape Metro area. Of these, approximately 23% were co-located in another district.

**FIGURE 1:**
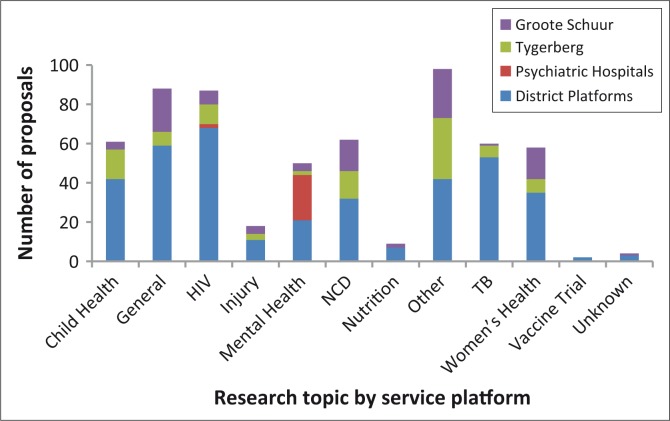
Research topics by service platform, Western Cape, January 2011 to August 2012. NCD, Non-communicable disease; TB, Tuberculosis.

Figure 1 summarises the topics for health research in the province. Combined, HIV (87 projects) and TB (60 projects) comprised the largest single category (28%) for health research in the province. Injury was the topic for only 4% of research projects in the province. Whilst mental health was the topic of 10% of all research in the province, less than half of all mental health projects were located at district level; the majority of studies were in specialised mental health hospitals. A subanalysis at one of the tertiary hospitals showed that 29% of studies approved were for drug trials and these were predominantly concentrated in non-communicable disease (NCD) categories (cardiac disease, Chronic Obstructive Pulmonary Disease, seizures, diabetes, cholesterol and oncology trials).

**FIGURE 2:**
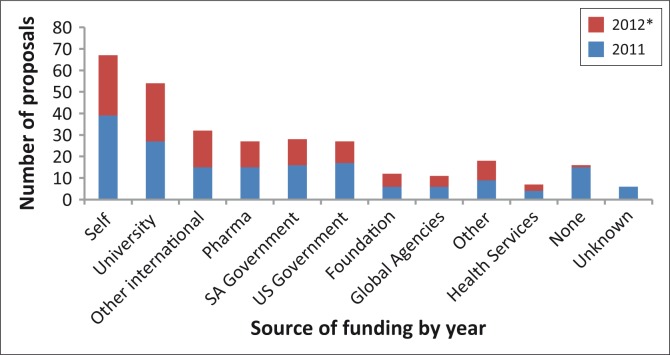
Source of funding for health research on the District platform, Western Cape, January 2011 to August 2012. *, Dataset for 2012 comprises eight months only – January to August 2012.

Researcher affiliation reflected local higher education or research institutions, with University of Cape Town (UCT) (36%), University of Stellenbosch (25%) and University of the Western Cape (10%) being the most frequent. A small number of projects (2%) reported an international researcher as principal investigator. The most common category for source of funding was researchers who reported being ‘Self-funded’ (57 projects or approximately 22%) and ‘University-funded’ (54 projects or approximately 17%). Projects funded by the pharmaceutical industry, the SA government, the US government and other international sources were all more-or-less equally frequent, comprising about 9% to 11% of projects.

**FIGURE 3:**
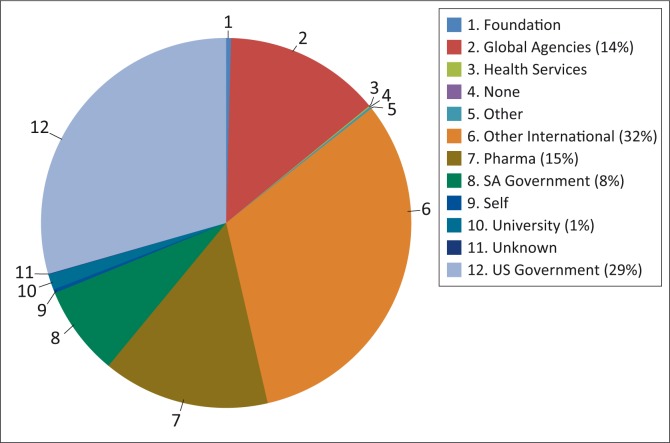
Total budget of health research funding on District Health platform, Western Cape, January 2011 to August 2012, by funding source category.

**FIGURE 4:**
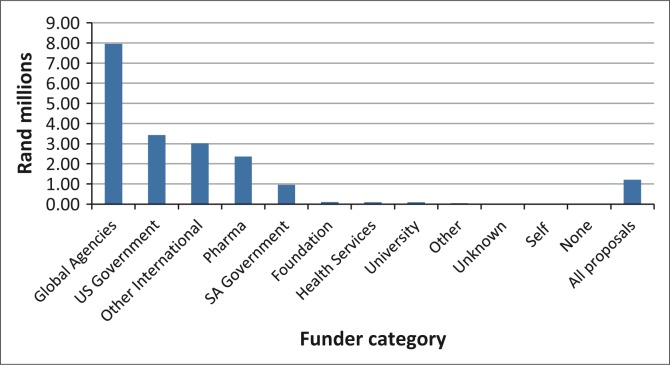
Average budget in R millions per project*, by funder category. *, Data on budgets missing for ∼30% of proposals, including > 50% pharma, global agencies.

Figures 3 and 4 summarise the size of research funding by funding source. Based on the 72% of projects for which budgetary details were provided, the largest categories of funder were ‘other international’ (32%) and the ‘US Government’ (29%), followed by ‘Pharma’ (15%), ‘Global Agencies’ (14%) and the ‘SA Government’ (8%). Of the SA government funding, the bulk came directly from government departments, particularly the Department of Health (88%), with relatively little coming via the two main national Research Councils (National Research Foundation 6% and Medical Research Council 4%). A single contribution from the Department of Health to the *South African National Health and Nutrition Examination Survey (*SANHANES) comprised more than 75% of all government funding. Without this contribution, government funding would have comprised about 2% of all health research funding on the district platform in the province.

In total, of the 72% of projects that reported budgets with sufficient detail to compute, a total of $29.2 million was spent on health research projects on the provincial health department district platform in the province over the 20 months for which budgetary data were available.

Figure 4 shows that funding in projects whose sources were ‘US Government’ or ‘Global Agencies’ were very large grants. Average budget per project for Global Agencies was close to $800 000 and that for US government about $340 000. By contrast, the average grant from SA government sources was about $95 000. Grants from the largest funders (Global Agencies, US Government, Other International) tended to focus on TB (37%), HIV (33%) and, less so, on women's health (11%) and child health (18%). Pharmaceutical funding included TB (14%), but was mostly directed at NCDs (30%) and other health conditions (23%). None of the largest funders supported injury-related research and only 4% of grants from these large funders were for mental health.

## Discussion

In contrast to developed countries,^16–19^ where health research spending is more-or-less in line with *national* BoD priorities, the findings in the Western Cape suggest a weak correlation with *local* BoD priorities. Whilst HIV and TB are undoubtedly critical contributors to BoD, the dwarfing of other areas of research in the province is striking – about 65% of all funding in the province was for projects related to HIV or TB or both. It is evident that research activities appear to be linked to donor funding availability focused on HIV and TB, rather than specific local priorities. This is a problem reported commonly in developing countries, where donor agendas have set the pattern of research.^8, 20, 21^ The mismatch between BoD and what research is taking place is concerning. For example, whereas the Western Cape suffers a very high burden from trauma, very little funding is directed at research addressing trauma and most of what is done on trauma is supported by SA government sources and smaller funders. Similarly, very little mental health research appears to be supported outside of psychiatric hospitals in the province. The lack of research in the nutrition field and the focus in NCD research on drug trials is another concerning aspect. Good nutrition is a key achievement of MDG goals 1 and 4 and NCDs continue to contribute a huge component of the provincial and national BoD, as well as placing a huge strain on the health services. Equally, the dearth in health systems research is a matter for concern as this denies the country empirical evidence upon which health decision makers can make judgement on how well the system is addressing the BoD, as well as what areas need attention.

This neglect of a certain area is not unique to the Western Cape or South Africa.^8^ For example, criticisms have been made regarding the neglect of mental health research in the UK.^22^ A review of US funding through the National Institutes of Health found that both mental health (as in depression) and injuries were underfunded areas compared with their US BoD;^23^ and both these morbidity categories were also noted as being underfunded in an Australian review of the extent to which health research funding followed the pattern of national with the burden of disease.^17^

Finally, both the quantum and pattern of research funding are potentially problematic. On the one hand, approximately US$30 million was spent on health research in the DHS. If one extrapolated to include studies which did not report funding details and studies in the health services elsewhere on the platform, the true figure may well be in excess of $50 million. This is substantial research expenditure and one may well ask whether the potential from this substantial investment in reduction of the BoD and improvement in quality of care is being realised.^24^ At the same time, the very low proportion of research funding in the province sourced from government funding structures is a matter for deep concern. About 8% of all research in the province receives SA government funding, with the bulk of research funding coming from international sources in the north. It is hard to imagine how local and national priorities can be met if 90% of research funding comes from outside sources, even with the best-intentioned forms of international aid. By contrast, there is evidence that Health Ministries in Europe take the lead in shaping and funding public health research in their countries.^18^

Calls have been made in international forums to increase national funding for health research such that at least 2% of the national health budget is allocated to health and development.^8, 25, 26^ Such a step will no doubt enable greater local determination of what kind of research is undertaken. However, it is sobering to think that such an increase would require a five-fold increase in funding in South Africa from the current allocation of 0.38% of the health budget for research.^3^ Whether such a massive investment is feasible and whether it will be done in such a way as to enable local priorities to be set and met remains to be seen.

Nonetheless, despite the likely difficulties, it can also been argued that SAs have a right to enjoy the benefits of research, a right which, by implication, imposes on the state an obligation to invest, to the maximum extent possible, in supporting scientific and technological advancement.^27^ Indeed, the right to benefit from scientific progress is contained in the International Covenant on Social, Cultural and Economic Rights (ICESCR), a covenant which South Africa signed in 1994 and agreed to ratify in 2012.^28^ Increased support and funding for health research, identified as a key action to strengthen the SA health system,^29^ would be entirely consistent with the cabinet's commitment to domesticate provisions of the ICESCR and with our Constitution's commitment to advancing the socio-economic rights of all who live in South Africa.

In addition to increased funding, we should also advocate for measures to strengthen the culture of using evidence for decision making, which is generally weak in the health system at present. For research to be of maximum benefit, there needs to be improved feedback of research findings by researchers to the services and to communities, as well as continuous review of research findings by the services in order to identify potential issues for action and translation of evidence into policy and programmes. Current plans to establish a national database of health research in South Africa being steered by the Health Systems Trust (HST) for the Department of Health will contribute to strengthening the health system's capacity to best utilise research findings for implementation.

There are a number of caveats to this study. Firstly, health research occurring off the service platform does not make its way into this database since it does not require health service permission. It is possible that much research in trauma and nutrition might fall into this category and may explain partly the dearth of these topics in the provincial database. Secondly, the database was not complete for all sources, limiting the detailed cross-tabulations regarding size of budget and researcher institution presented here to the Provincial DHS database. Thirdly, information supplied by investigators was often missing or inaccurate, particularly related to financial data and funding source. Lastly, in extrapolating from data provided to reach a minimum figure of $50 million spent on health research in the Western Cape over a 24-month period, there may be a number of variables that make this figure either an under- or over-estimate. The funding profile of projects for which financial data were missing may have been different from those that were reported and there may have been some double counting if the principal investigator applied to more than one authority. Nonetheless, the estimate remains a sizeable figure for health research in the province and points to the need to find mechanisms with which to maximise the added value for the health of the population of the Western Cape from such a large investment of funding.

## Conclusion

This audit provides the first provincial report on health research conducted in South Africa. It confirms that considerable resources are invested in research in the province, but that there are key gaps in what is funded. Neglected areas such as mental health, malnutrition and trauma are important contributors to BoD but, presumably because they are not attractive to large donors, do not receive levels of funding commensurate with local need. The National Health Summit in South Africa in 2011 made seven key recommendations regarding the future of health research in this country. The findings of this review echo some of these calls, particularly the need for increased national funding for health research. If South Africa is serious about wanting to shape the portfolio of research for development and evidence-based decision making, the state needs to increase substantially its funding for health research. Otherwise the country's pressing health needs and challenges will not be adequately addressed and research direction will remain determined by the interests of external agencies which may not always be consistent with national and local health priorities. We believe the situation may be even more extreme in other African countries, where national support for health research is even more constrained.
